# Association between physical exercise and innovative behavior among university students: the mediating role of learning engagement

**DOI:** 10.3389/fpsyg.2026.1749634

**Published:** 2026-02-13

**Authors:** Meiyuan Yang, Ye Lin, Dongmei Liu, Wanpeng Zhen, Yongchao Jin, Sihan Yan, Yang Li, Jianwei Chen

**Affiliations:** 1 College of Architecture and Civil Engineering, North China University of Science and Technology, Tangshan, China; 2 College of Education, St. Paul University Manila, Manila, Philippines; 3 Rear Services Center, North China University of Science and Technology, Tangshan, China; 4 School of Science, North China University of Science and Technology, Tangshan, China; 5 School of Economics and Management, North China University of Science and Technology, Tangshan, China

**Keywords:** innovative behavior, learning engagement, mediating role, physical exercise, student

## Abstract

**Purpose:**

This study examined the relationship between physical exercise and innovative behavior in university students, with a particular focus on the mediating role of learning engagement. The findings aimed to provide insights for fostering innovative talents and enhancing the quality of talent development in higher education.

**Methods:**

By means of convenient sampling, a total of 1,176 students were recruited from a comprehensive university in North China to participate in the online survey. This study employed the physical exercise scale, the learning engagement scale, and the innovative behavior scale as the main tools. The data were analyzed using SPSSAU, and descriptive statistics, correlation analysis, regression analysis and tests were employed to examine the mediating role of learning engagement.

**Results:**

(1) There was no significant gender difference in learning engagement and innovative behavior among college students (*p* > 0.05); In terms of physical exercise, there was a significant gender difference (*p* < 0.05). (2) Physical exercise is positively correlated with college students’ innovative behavior (*r* = 0.435, *p* < 0.01), positively correlated with learning engagement (*r* = 0.524, *p* < 0.01), and learning engagement was positively correlated with innovative behavior (*r* = 0.314, *p* < 0.01). Physical exercise has a significant direct effect on innovative behavior, explaining 72.6% of the variance. Learning engagement plays a mediating role between physical exercise and innovative behavior, with an effect value of 27.4%.

**Conclusion:**

(1) The research results revealed a significant interrelationship among physical exercise levels, learning engagement and innovative behaviors among college students. (2) Physical exercise was a significant predictor of innovative behavior and further indirectly influences it through the mediating role of learning engagement.

## 1 Introduction

As China’s higher education enrollment continued to expand (Fan, 2020; Feng and Xia, 2022), university students have become a pivotal force in advancing the nation’s “innovation-driven development strategy.” According to the latest data released by the Ministry of Education, the gross enrollment rate of higher education in China had reached 60.8%, marking that higher education had entered the stage of popularization. This meant that how to continuously promote university students’ participation in innovative activities and enable them to demonstrate higher levels of creativity and more innovative behaviors had become a top priority in the current phase of China’s higher education reform (Gu et al., 2017; Lei et al., 2020). In 2019, the “China Education Modernization 2035” plan explicitly stated that the world today was in a period of major development and transformation, and to achieve educational modernization, it was essential to persistently implement the innovation-driven development strategy and advance China’s position as a strong talent nation (Central Committee of the Communist Party of China and State Council of China, 2019; Hu and Zhao, 2023). From the perspective of behavioral science, the innovative behavior of students referred to their generation of unique ideas, active exploration of new methods, willingness to take risks, and ultimately achieving valuable innovative results during the research process (Bjornali and Anne Storen, 2012; Chen et al., 2018). University students’ innovative behavior manifested through knowledge exploration, problem-solving, and creative initiative, served as a critical indicator of their innovative potential (Kim et al., 2018; Dai et al., 2022). Therefore, an in-depth exploration of the influencing factors and mechanisms of college students’ innovative behaviors was of great significance for the development of contemporary higher education.

In 1988, Amabile first proposed the concept of innovative behavior, stating that innovative behavior referred to an individual actively putting forward new ideas, new viewpoints or new solutions to problems during the work process (Amabile, 1988). Scott and Bruce (1994) held that innovation behavior begins with problem identification, followed by the generation of innovative ideas, problem-solving methods, or solutions that gain recognition from others, ultimately achieving the “productization” of these innovative ideas. Janssen emphasized that innovation requires not only the generation of ideas but also their effective application (Janssen, 2000; Janssen, 2004). In educational settings, this entailed seeking creative solutions and support to turn ideas into reality (Saengpanya, 2018; Scott and Bruce, 1994). Accordingly, this study defined innovative behavior as students’ ability to generate, refine, and implement ideas to solve problems and improve learning outcomes (Saengpanya et al., 2025). In the field of higher education research, numerous scholars have conducted in-depth discussions on innovative behavior from multiple perspectives and based on different theoretical frameworks (Kwon and Kim, 2020; Montani and Staglian’o, 2022; Saleem et al., 2015; Yu et al., 2023). For example, Self-Determination Theory (SDT) emphasized the central role of intrinsic motivation in innovative behavior, which was considered the key driver for individuals to engage in innovative activities (Deci and Ryan, 2000). This implied that when students had a strong interest in learning and innovation itself, along with self-driven motivation, they were more likely to demonstrate innovative behavior. In contrast, social cognitive theory highlighted the impact of the reciprocal interaction between personal beliefs and the environment on behavior (Bandura, 2001). Bandura’s triadic reciprocal determinism posited that an individual’s behavior, cognition, and environment interacted and influenced one another (Bandura, 2001). In educational contexts, this meant that students’ innovative behavior was not only driven by their own innovative beliefs, such as self-efficacy, but was also positively or negatively influenced by environmental factors such as teachers and peers. These theories and perspectives provided rich research paths for understanding the generation, development and influencing factors of college students’ innovative behaviors (Xu et al., 2024).

Physical exercise referred to the practical process of enhancing physical fitness, promoting physical and mental health, improving physiological functions and achieving all-round development through systematic physical activities (Xi, 2004). Physical exercise was one of the fundamental ways to achieve the goals and tasks of physical education. It encompassed a series of organized and planned forms of sports practice, aiming to enhance an individual’s health level and quality of life through the implementation of specific sports events (Caspersen et al., 1985). According to the theory of neuroplasticity, physical exercise could reshape the structure and function of the hippocampus to a certain extent, leading to changes in the number and connectivity of synapses, and ultimately improving mood and enhancing memory (Man and Wei, 2021). Meanwhile, physical exercise could activate the prefrontal cortex (PFC), parietal cortex and posterior cingulate cortex (PCC), thereby improving all aspects of cognition and performance (Hillman et al., 2008). This neural mechanism regulatory effect could also significantly enhance an individual’s information encoding and extraction efficiency, and strengthen the overall performance of the memory system (Kayani et al., 2018). In addition, college students who had reached the internationally recommended moderate to high-intensity exercise level showed a lower incidence of academic burnout (Gerber et al., 2013), thus, it had a significant promoting effect on the improvement of academic performance (Sun et al., 2025). Existing empirical studies further confirmed that physical exercise had a significant promoting effect on the development of individual creativity (Sun et al., 2025; Büning et al., 2020; Rodríguez-Negro and Yanci, 2021). As Zhao et al. found through experimental research, short-term aerobic exercise could simultaneously enhance the convergent thinking and divergent thinking abilities of the subjects (Zhao et al., 2022).

Learning engagement referred to the degree of energy commitment, cognitive participation and emotional concentration that students demonstrated in academic activities (Skinner et al., 2008). As an important indicator for measuring learning quality, its characteristic was that students maintain a state of abundant vitality, deep participation and high concentration during the learning process. Gardner (1993) and other scholars’ research on highly creative individuals found that such people tend to invest a great deal of time and energy in their professional fields, demonstrating a completely immersed working state. Taking Einstein as an example, he often spent several days delving into the same scientific issue. Such innovators generally possessed the typical characteristic of “constantly thinking about work”. The research of Seibert et al. further indicated that proactive individuals were more inclined to promote innovation by constantly improving their working methods (Seibert et al., 2001). They tended to generate novel ideas more easily, continuously enhanced their performance levels, and eventually achieved professional breakthroughs. An empirical study conducted by domestic scholar (Han, 2014) on 225 academic master’s students also confirmed that learning engagement had a significant positive predictive effect on the innovation ability of postgraduate students. Senior students often had richer knowledge reserves and higher learning autonomy (Wang, 2006). Their cognition and motivation for innovative behavior might also have differed from those of junior students. All these studies indicated that learning engagement was a key antecedent variable for predicting innovative behavior.

Although existing studies had explored the influencing factors of innovative behavior from multiple perspectives such as individual cognition, personality traits, teaching environment, and social support, the role of physical exercise, a fundamental and interventionable factor, in the research system of innovative behavior had not yet received sufficient attention and in-depth explanation. Physical exercise not only concerned students’ physical health, but had also been proven to significantly influence cognitive functions (such as attention, memory, and executive function), emotional states (such as reducing stress and enhancing positive emotions), and psychological resilience, all of which were essential psychological resources for stimulating and maintaining innovative thinking and behavior. Learning engagement, as a positive and complete psychological state full of vitality, concentration and dedication demonstrated in academic activities, served as the core bridge connecting students’ external behaviors with their internal cognitive emotions. We inferred that regular physical exercise might enhance students’ energy levels, improve their moods and strengthen their self-regulation abilities, thereby promoting their commitment to learning tasks (vitality, concentration and dedication). A high level of learning commitment implied deeper information processing, broader knowledge connection and more persistent problem exploration. This was precisely the fertile soil for nurturing innovative ideas and putting them into practice (that is, innovative behavior).

Based on the self-determination theory, physical exercise helped enhance an individual’s intrinsic motivation and learning engagement, thereby promoting the occurrence of innovative behaviors. A study on college students’ physical exercise behavior also found that gender significantly affected college students’ exercise motivation, self-efficacy and physical activity level. Men usually had a higher sense of exercise self-efficacy than women (Trost et al., 2002). This meant that students of different genders might have differences in learning motivation and self-regulation ability, thereby affecting their learning engagement and innovative behavior. Although existing studies had, respectively, explored the relationship among physical exercise, learning engagement and innovative behavior, there was still a lack of an integrated mediating model to systematically reveal “how physical exercise promoted innovative behavior by influencing the psychological state (i.e., learning engagement) of college students during their core academic process.” Based on self-determination theory and social cognition theory, this study focused on exploring the promoting effect of physical exercise on the innovative behavior of college students by enhancing learning engagement. The research resulted not only provide a new theoretical perspective for understanding the intrinsic mechanism between physical exercise and innovative output, but also offered empirical support for universities to systematically cultivate innovative talents by integrating physical and mental health education with academic investment. Based on this theoretical framework, this study proposed the following hypotheses:

*Hypothesis 1*: There is a positive correlation between physical exercise and innovative behavior among college students.

*Hypothesis 2*: Learning engagement is positively associated with innovative behavior among college students.

*Hypothesis 3*: There is a positive relationship between the physical exercise and learning engagement among college students.

*Hypothesis 4*: Learning engagement mediates the relationship between physical exercise and innovative behavior among college students.

## 2 Participants

Limited by the research period and implementation conditions, this study selected 1,176 college students from a comprehensive university in North China as the research subjects by using the convenience sampling method. In this study, a “comprehensive university” referred to a full-time general higher education institution that encompassed multiple disciplines such as natural sciences, engineering technology, humanities and social sciences, and management sciences, with undergraduate and postgraduate education as its primary responsibilities. In this study, the sample size was calculated based on the finite population cross-sectional formula $n=\frac{{\left(\frac{Z_{\alpha }⋆\sigma }{\delta }\right)}^{2}}{1+{\left(\frac{Z_{\alpha }⋆\sigma }{\delta }\right)}^{2}/N}\;$ , and considering a 5% rate of invalid questionnaires, it was determined that the sample size should be at least 630 participants. Although this method could ensure the efficiency of data collection, it might impose certain limitations on the generalization scope of research conclusions. Subsequent research could adopt more representative sampling methods, such as stratified sampling, to enhance the heterogeneity of the sample and the validity of the inference.

This study used the professional online research platform “Wenjuanxing” to distribute questionnaires. The target group was invited to participate in the survey through link sharing. The data collection period was from October 1st to 7th, 2025, and a total of 1,200 questionnaires were distributed. The entire process strictly adhered to the principle of voluntary participation. Before conducting the formal test, the purpose of the research should be clearly explained to all participants and they should be informed that their responses will be strictly confidential. Only those who agreed could complete the questionnaire. In terms of identifying invalid questionnaires, a strict screening process were implemented, including eliminating questionnaires with overly short answering times (less than 120 s) or those with a large number of missing items, excluding questionnaires with contradictory answers or obviously perfunctory responses, using trap questions to eliminate those who had not read the questions carefully, and deleting repeated submissions with the same IP address or highly similar answering patterns. After review, 24 invalid or incomplete questionnaires were excluded, and ultimately 1,176 valid questionnaires were obtained, with an effective recovery rate of 98%. The gender composition of the sample was 303 males (25.77%) and 873 females (74.23%). This sample was characterized by a relatively high proportion of females and a relatively high proportion of lower-grade students. This was because Wenjuanxing platform, based on the core quota requirements set by researchers (with the main target group being students from a comprehensive university in Hebei Province), conducted questionnaire push and data collection in the active user database it covered. The final participation was entirely determined voluntarily by the users, and the researchers did not conduct targeted interventions for specific institutions or individuals. Despite the above-mentioned distribution differences, this did not diminish the value of this study in testing the core mediating mechanism. In the questionnaire survey, a demographic information collection module were specially set up, which included a single-choice question on “Whether you are an only child,” with options including “Yes” and “No.” The specific demographic characteristic distribution is shown in Table 1. In Table 1, the column “quorum” referred to the specific number of people corresponding to each categorical variable (such as gender, grade, only child status, etc.) among the 1,176 valid samples finally included in the analysis after data cleaning.

**Table 1:** Distribution of demographic variables among students.

Variant	Form	Quorum	Percentage (%)
Sex	Male	303	25.77
	Female	873	74.23
Grade	First-year student	452	38.44
	Second-year student	345	29.34
	Third-year student	222	18.88
	Fourth-year student	148	12.59
	Fifth-year student	9	0.77
Plans after bachelor’s degree graduation	Continue education	519	44.13
	Work	277	23.55
	Other	34	2.89
	Not sure	346	29.42
Academic performance	Superior	145	12.33
	Above average	383	32.57
	Average	499	42.43
	Below par	114	9.69
Are you (or have you been) a class or student club officer?	Yes	524	44.56
	No	652	55.44
Number of students in the class	1–30	1,030	87.59
	31–60	134	11.39
	>61	12	1.02
Only child status	Yes	266	22.62
	No	910	77.38
Total		1,176	100.0

## 3 Methods

The “Physical Exercise Scale,” “Innovative Behavior Scale” and “Learning engagement” adopted in this study were all mature scales that had been widely used in related research at home and abroad and whose reliability and validity had been verified. To test its applicability and measurement quality in the current research sample, the following measurement characteristic assessment procedures were implemented: Firstly, in terms of content validity, two experts in the field of physical education were invited to review the degree of consistency between the scale items and the research background as well as the clarity of expression. The results showed that all the entries had good relevance and clarity. Secondly, reliability and validity tests were conducted based on the data obtained from this survey. Reliability analysis showed that the Cronbach’s α coefficients of each scale were all higher than 0.93, indicating a high degree of internal consistency. The structural validity was evaluated through confirmatory factor analysis. All the model fitting indicators met the ideal standards (CFI > 0.90, TLI > 0.90, RMSEA < 0.1), indicating that the original scale structure had good adaptability in this study.

### 3.1 Physical exercise scale

The measurement tool for the variable of physical exercise was the physical exercise assessment questionnaire for college students developed by Wu (2016). This scale consisted of 8 items, which were used to measure two dimensions: “physical exercise commitment” and “physical exercise persistence.” Physical exercise commitment: this dimension primarily measured the degree to which an individual identified with, values, and feels attached to physical exercise on a cognitive and emotional level. Through four items, it captured the student’s willingness to regard physical exercise as an important component of daily life, their emotional desire to participate in exercise, and their inherent tendency to prioritize exercise even when faced with competing activities. Physical exercise persistence: this dimension mainly measured the stability and perseverance of an individual in engaging in regular, long-term physical exercise behaviorally. Through four items, it reflected the frequency and consistency of students’ actual participation in physical exercise, as well as their behavioral ability to maintain exercise habits in the face of difficulties. All items were evaluated using a 5-point Likert scale (1 = strongly disagree, 5 = strongly agree). A total average of all item scores were calculate as the comprehensive indicator of an individual’s physical exercise level. In this measurement, the Cronbach’s α coefficient of this questionnaire was 0.947, indicating that it had a relatively high internal consistency.

### 3.2 Innovative behavior scale

The measurement of innovative behavior adopted the scale developed by Wang et al. (2022). This scale had a one-dimensional structure and consisted of 6 items. It used a 5-point Likert scoring system ranging from “1 = strongly disagree” to “5 = strongly agree.” The higher the total score of the scale, the higher the level of an individual’s innovative behavior. In this study, the internal consistency reliability of this scale was good, with a Cronbach’s α coefficient of 0.933.

### 3.3 Learning engagement

The measurement of learning engagement employed the scale developed by Schaufeli et al. (2002). This scale consisted of 17 items, covering three dimensions: motivation, energy and concentration. It adopted a five-point Likert scoring system ranging from “1 = strongly disagree” to “5 = strongly agree.” The higher the total score of the scale, the higher the degree of learning commitment of the students. The original scale had robust structural validity, and the internal consistency reliability of the total scale and each dimension was all higher than 0.75. In this study, the Cronbach’s α coefficient of the total scale was 0.971, indicating good reliability.

### 3.4 Statistical analysis

The SPSSAU statistical analysis platform was used for data processing in this study. Firstly, the deviation of the commonly used methods was tested by using the Harman single-factor test. Subsequently, descriptive statistics, independent sample *t*-tests, and one-way analysis of variance (ANOVA) were used to present sample characteristics and overall differences. On this basis, the pearson correlation analysis method was adopted to preliminarily explore the pairwise relationships among the three core variables of physical exercise, learning engagement and innovative behavior.

In the model, physical exercise was taken as the independent variable (X), innovative behavior as the dependent variable (Y), and learning engagement as the mediating variable (M). The significance test of the mediating effect of learning engagement was conducted using the Bootstrap sampling method (repeated sampling 5,000 times) (Hayes, 2015; Hayes, 2018), and its 95% confidence interval was calculated.

## 4 Results

### 4.1 Common method bias test

To evaluate the common method bias, this study conducted a Harman single-factor test (Zhou and Long, 2004). The results showed that the first unrotated common factor explains 39.16% of the total variance, which was lower than the 40% criterion, indicating that the common method bias did not show a significant impact in this study.

### 4.2 Descriptive statistics and correlation analysis

In terms of data analysis, this study mainly focused on the relationship among various variables at the total score level and did not explore their detailed dimensions. Therefore, the mean values of physical exercise, learning engagement and innovative behavior were calculated, and a Pearson correlation analysis was conducted. According to the widely applied standard in the social sciences proposed Lachenbruch and Cohen (1989), an absolute correlation coefficient of around 0.10 is generally considered a weak correlation, around 0.30 a moderate correlation, and 0.50 or above a strong correlation. In this study, there was a significant positive correlation between physical exercise and innovative behavior (*r* = 0.600, *p* < 0.01), and there was also a significant positive correlation between learning engagement and innovative behavior (*r* = 0.542, *p* < 0.01) (see Table 2). Meanwhile, there was a significant positive correlation between physical exercise and learning engagement (*r* = 0.524, *p* < 0.01). This indicated that there were statistically significant strong correlations among the variables.

**Table 2:** Mean, standard deviation, and correlation analysis of physical exercise, innovative behavior and learning engagement.

	Physical exercise	Innovative behavior	Learning engagement
Physical exercise	1		
Innovative behavior	0.600**	1	
Learning engagement	0.524**	0.542**	1
*M*	3.376	3.716	3.349
SD	0.907	0.766	0.913

**p < 0.01.

### 4.3 Independent samples *t*-test and ANOVA

This study used an independent sample *t*-test to investigate the influence of gender on each variable (the results are shown in Table 3). The results of Levene’s homogeneity of variance test showed that innovative behavior and learning engagement exhibited non-homogeneous variance (*p* < 0.01), thus non-homogeneous variance data were needed. In contrast, there was no significant difference in physical exercise (*p* > 0.05). In terms of mean comparison, two independent sample t-tests were conducted, analyzing the imbalanced sample (303 males and 873 females) and the balanced paired sample (303 males and 380 females) respectively. The results showed that there were statistically significant differences (*p* < 0.01) in the physical exercise levels of college students of different genders, whether in the original sample or the balanced sub-sample (see Supplementary Table S1), and the results were consistent. However, in terms of innovative behavior and learning engagement, the average differences between genders did not reach a significant level. In conclusion, gender only had a significant impact on the variable of physical exercise. This indicates that an unbalanced gender ratio in the sample does not have a substantial impact on the validity of gender comparisons, and the research results are robust.

**Table 3:** Independent samples *t*-test for sex.

		Levine’s test of variance equivalence		Mean equivalence *t*-test	
Grouping variable	Implicit variable	*F*	*p*	*t*	*p*
Gender	Physical exercise	2.325	0.128	4.171	0.000**
	Innovative behavior	12.327	0.000**	−1.899	0.058
	Learning engagement	8.661	0.003**	−1.170	0.243

**p < 0.01.

To investigate the influence of different academic stages (such as freshmen to seniors) on various variables, this study conducted one-way analysis of variance (ANOVA), and the results were shown in Table 4. The results indicated that there were no significant differences in innovative behavior and learning engagement among different academic stages (*p* > 0.05). However, there were significant differences in the level of physical exercise among different academic stages.

**Table 4:** ANOVA for grade.

Grouping variable	Implicit variable	Mean square	*F*	*p*
Grade	Physical exercise	14.929	17.393	0.000**
	Innovative behavior	2.41	3.118	0.078
	Learning engagement	1.82	1.185	0.277

**p < 0.01.

### 4.4 Mediating effect of learning engagement in college students’ physical exercise and innovative behavior

**Figure 1:**
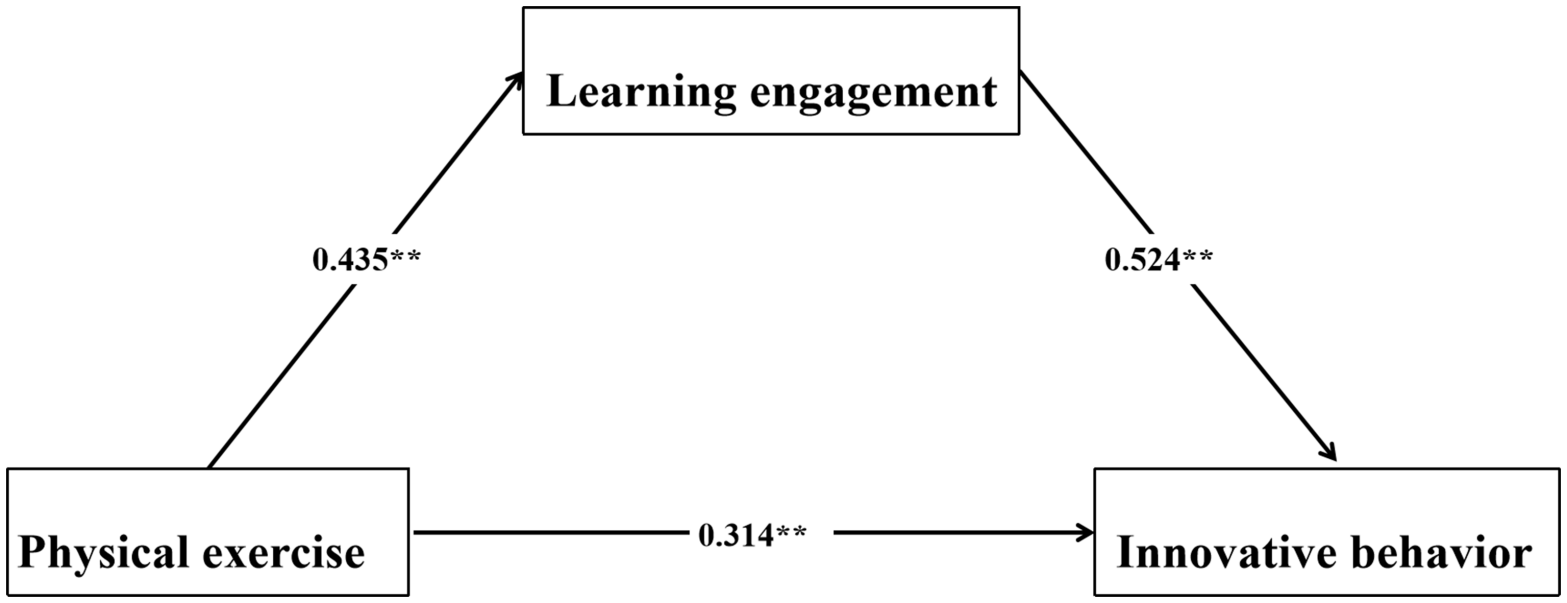
Pathways of physical exercise influence on innovative behavior. **p < 0.01.

To examine the mediating role of learning engagement, the significance test was conducted using the Bootstrap method (with 5,000 repeated samplings), and the results supported its significant mediating role (*p* < 0.01). The path coefficient of the models all reached the statistically significant level, the specific path relationship was shown in Figure 1. Specifically, the direct effect of physical exercise on innovative behavior was significant. Meanwhile, the mediating effect value of learning engagement was 0.139, and its 95% Bootstrap confidence interval was [0.129, 0.207], excluding 0, indicating that this indirect effect was significant. This indirect effect accounted for 27.40% of the total effect (0.507). The results showed that more than a quarter (27.4%) of physical exercise had a positive impact on college students’ innovative behavior by enhancing their learning engagement.

The above results indicated that physical exercise could not only directly predict the innovative behavior of college students, but also had an indirect impact through the mediating path of learning engagement. Specifically, more than a quarter (27.4%) of the promoting effect was achieved by enhancing students’ learning engagement (such as improving attention, deepening cognitive processing, etc.). This indicated that guiding students to continuously and actively participate in learning activities was a key internal mechanism for physical exercise to effectively promote the development of their innovative behaviors (Table 5).

**Table 5:** Explanatory table for total, direct, and indirect effects.

Parameters	Estimates of value	Lower limit	Upper limit	*p*	Effect proportion/%
Indirect effect	0.139	0.129	0.207	0.000	27.4
Direct effect	0.368	0.325	0.410	0.000	72.6
Total effect	0.507	0.468	0.545	0.000	100

## 5 Discussion

This study revealed the intrinsic mechanism by which physical exercise promoted college students’ innovative behavior by enhancing learning engagement, and confirmed the key mediating role of learning engagement between the two variables. This discovery was of great value in stimulating students’ enthusiasm for participating in sports, cultivating their innovative ability and promoting the development of their comprehensive quality. At the theoretical level, this study deepened the understanding of the formation mechanism of innovative behavior, expanded the application boundaries of the student engagement theory, and provided a new perspective for interdisciplinary research in positive psychology and education. At the practical level, the results highlighted the dynamic synergy among physical exercise, cognitive engagement and innovative performance. It provided a theoretical basis and practical path for educators to stimulate learning motivation and cultivate innovative talents by systematically promoting students’ physical exercise.

### 5.1 Sex differences in physical exercise

The results of this study showed that there were significant gender differences in college students’ participation in physical exercise. This phenomenon could be explained from three dimensions: social culture, psychological motivation and institutional environment. Firstly, the traditional socialization process of gender roles had a profound impact on participation in physical exercise. Men were usually encouraged to participate in structured and highly competitive sports activities from an early age, which made physical exercise more easily integrated into their daily lives and formed stable habits (DeWolfe et al., 2019). In contrast, women often faced the normative expectations of society for “femininity,” while lacking the effective incentive of high-intensity physical exercise. These factors jointly affected the establishment of their sports identity and the sustainability of their exercise behavior. Secondly, there were systematic gender differences in the motivation for participation in physical exercise and project preferences. Men generally tended to choose sports that required teamwork, strong competitiveness and high intensity (such as basketball, football, etc.). Such activities usually enjoyed higher social recognition and participation convenience in campus culture. Women, on the other hand, preferred personalized and goal-oriented activities that focused on physical and mental health (such as yoga, Pilates, jogging, etc.), and their participation motivations were more derived from personal well-being rather than competitive needs (Huang et al., 2021). Finally, there might be an unconscious gender bias in the institutional design and environmental construction of campus sports. The current planning of sports facilities, the organization of school leagues and the promotion of fitness programs were often more in line with the traditional male-dominated sports model. This structural imbalance might cause female students to feel neglected, limit their access to physical exercise opportunities that suited their own characteristics, and ultimately affect their long-term willingness to participate in sports and the sustainability of their behavior (Bao, 2025).

### 5.2 Direct effects of university students’ physical exercise on innovative behavior

The results of this study indicated that physical exercise exerted a significant positive predictive effect on the innovative behavior of college students (direct effect value = 0.368), and Hypothesis 1 was supported. This finding was consistent with the existing research conclusions. Physical exercise could not only improve the physical state, but also contributed to enhancing cognitive function and mental health level, both of these aspects played a key role in the innovation process (Gong and Yang, 2023; Yu and Zhang, 2019). According to the cognitive stimulation theory, physical exercise could promote the increase of cerebral blood flow, stimulate neurogenesis, and effectively enhance executive functions including cognitive flexibility, working memory and inhibitory control (Liu et al., 2025). These advanced cognitive abilities were precisely the core basis of creative thinking. In the challenging academic environment of universities, students often encountered a high degree of stress and cognitive fatigue, which might inhibit their creative performance. Regular physical exercise had important functions of relieving stress and improving emotional state, and could create more favorable psychological conditions for the gestation and exploration of innovative ideas (Lin et al., 2022). Existing empirical studies had also proposed that physical exercise had a positive impact on stimulating individual creative potential and improving problem-solving ability (Zhao et al., 2022). Based on the existing research, this study further verified the direct predictive relationship between physical exercise and innovative behavior, accumulating new empirical evidence in this field. This study also provided important inspirations for promoting innovative behavior among college students at the same time: By systematically promoting college students’ participation in physical exercise, a solid physical and psychological foundation could be laid for their innovative behaviors.

Therefore, colleges and universities should have incorporated physical exercise into the innovative talent cultivation system and go beyond its traditional positioning as an entertainment activity. Specifically, it was suggested to promote from the following aspects: In terms of hardware construction, it was necessary to improve the layout of campus sports facilities, increase investment in multi-functional gyms, dance studios, outdoor sports areas and other venues, and create an open, inclusive, convenient and accessible sports exercise space. In terms of curriculum reform, a diversified physical education curriculum system should have been established, with the addition of distinctive projects such as fitness training, traditional ethnic sports, and outdoor expansion. The single skills assessment model should have been changed, and emphasis should have been placed on cultivating students’ interest in sports and their habit of persistence. In terms of cultural cultivation, it could create a campus cultural atmosphere that valued sports and encouraged innovation by holding subject-integrated sports competitions and creative sports events, and by leveraging campus media to publicize the empirical value of physical exercise in cognitive promotion and innovative performance.

### 5.3 Mediating effects of learning engagement

Based on the results of the mediating effect analysis, this study found that physical exercise had a dual mechanism of action on the innovative behavior of college students. On the one hand, physical exercise could directly and positively predict the performance of innovative behavior, indicating that improving the physical exercise level of college students could directly promote the development of their innovative ability. On the other hand, physical exercise also had an indirect impact through the mediating path of learning engagement, that is, physical exercise promoted innovative behavior by enhancing the level of learning engagement. This finding supported hypothesis H2, H3, and H4. According to the expansion-construction theory proposed by Fredrickson (2001), the positive emotional state stimulated by physical exercise could expand an individual’s cognitive-behavioral resources, enhance openness to new experiences and psychological resilience in dealing with challenges, and provide a necessary foundation for innovative behaviors. Empirical studies further confirmed that regular physical exercise was significantly associated with improved attention, enhanced vitality, and increased engagement in learning tasks, while sedentary behavior was negatively correlated with learning engagement (Liang et al., 2022), which provided empirical support for understanding the internal mechanism by which physical exercise affected innovative behavior through learning engagement.

According to the theory of conservation of resources (Hobfoll, 1989), individuals had an instinctive tendency to maintain and accumulate psychological resources, and physical exercise was precisely an effective way to obtain both physical and psychological energy. The cognitive resources and positive emotions stored through regular physical activities could provide necessary support for continuous and in-depth learning activities, thereby significantly enhancing the learning engagement level of college students. Meanwhile, existing studies consistently indicated that learning engagement was a key factor in predicting academic achievement and innovative performance (Zhu et al., 2011). A highly engaged learning state could promote the development of cognitive flexibility, enhance knowledge integration ability, and cultivate critical thinking qualities—these cognitive traits were precisely the core foundation for generating original ideas. In complex academic situations, high learning inputs were more inclined to fully immerse themselves in the problem-solving process, demonstrating stronger perseverance and interdisciplinary information integration capabilities. These behavioral characteristics were precisely important indicators of innovation output. Furthermore, the psychological capital accumulated through deep participation in learning, such as self-efficacy and achievement motivation, also provided crucial psychological support for students to propose and practice innovative solutions (Hsu and Chen, 2015; Wang et al., 2022). Based on this theoretical framework, this study verified the mediating mechanism of learning engagement between physical exercise and innovative behavior. The 27.4% mediating effect value not only quantitatively confirmed the connecting role of learning engagement, but also revealed the specific path by which physical exercise promoted innovative output by enhancing psychological resources. This discovery provided important enlightenment for the educational practice in colleges and universities: while encouraging students to maintain regular physical exercise, targeted intervention plans to enhance learning engagement should be designed, such as systematically cultivating college students’ innovative qualities through measures like optimizing curriculum design and creating supportive learning environments. This two-pronged strategy would maximize the promoting effect of physical exercise on innovative behavior, and provide an effective path for the cultivation of innovative talents.

Therefore, this study suggested that university administrators and educators should have fully attached importance to the intrinsic connection between physical exercise and learning engagement, and incorporated it into the innovative talent cultivation plan. Specifically, colleges and universities could set up structured physical exercise time in their daily teaching schedules, such as organizing 10 min of collective stretching, aerobic exercises or campus brisk walking and other micro-exercises during breaks, so as to transform physical exercise from an extracurricular supplement to an organic part of the academic ecosystem. This institutional arrangement helped to directly activate students’ cognitive states through physical activities, enhance their learning concentration, and thereby create conditions for innovative behaviors. Meanwhile, professional training for the student affairs team should have been strengthened. The relevant content of the mechanism of action of “physical exercise - learning engagement - innovative behavior” should have been incorporated into the training system of student counselors and academic mentors. Guided students to scientifically apply physical exercise strategies to optimize the allocation of attention, enhance learning energy, and ultimately improve academic innovation performance.

## 6 Conclusion

Based on the survey data of 1,176 college students, this study explored the intrinsic connection among physical exercise, learning engagement and innovative behavior. The results indicated that there were significant positive correlations among the three variables of college students’ physical exercise level, learning engagement degree and innovative behavior performance. In-depth analysis revealed that physical exercise could not only directly and positively predict the innovative behavior performance of college students, but also exerted an indirect promoting effect through the mediating variable of learning engagement. This discovery revealed the crucial bridging role of learning engagement in the relationship between physical exercise and innovative behavior, providing a new theoretical perspective for understanding the formation mechanism of college students’ innovative behavior.

## 7 Limitations

This study revealed the intrinsic connection among college students’ physical exercise, learning engagement and innovative behavior through empirical analysis. However, due to the limitations of research conditions, there were still several aspects that need to be improved.

Firstly, at the research design level, cross-sectional data was difficult to establish causal relationships among variables. Future research could adopt longitudinal tracking or experimental intervention methods. For instance, freshmen in universities could be selected as research subjects to conduct a three to four-year follow-up investigation, systematically examining the dynamic development trajectories and interaction mechanisms of physical exercise habits, learning engagement levels, and innovative behavior performance. This would help to build a clearer causal chain. Secondly, in terms of sample representativeness, this study mainly focused on students from regular undergraduate colleges. In the future, the sampling scope could be expanded to include students from higher vocational colleges, different geographical distributions, and diverse cultural backgrounds. By enhancing sample heterogeneity, the external validity of the research conclusions could be improved, enabling the research results to be more widely applicable to various types of higher education scenarios.

At the practical application level, based on the theoretical findings of this study, efforts could have been made in the future to develop systematic educational intervention programs. Specifically, a comprehensive intervention model that encompassed both sports promotion and learning reinforcement could have been established. In terms of sports promotion, measures such as setting up campus fitness challenges, fostering sports-related student associations, and optimizing sports venues and facilities could have been adopted to create a favorable campus sports culture atmosphere. In terms of learning reinforcement, active learning methods such as problem-oriented learning and project-based tasks could have been integrated into professional courses to stimulate students’ in-depth cognitive participation and cultivate their innovative thinking abilities. This two-pronged intervention strategy not only helped to verify the theoretical model of this study, but also provided practical and feasible solutions for universities to cultivate innovative talents, promoting the overall improvement of higher education quality.

## Data Availability

The original contributions presented in the study are included in the article/Supplementary material, further inquiries can be directed to the corresponding author.
