# Supplementary material for: Association between physical exercise and innovative behavior among university students: the mediating role of learning engagement
Source: Front Psychol. 2026 Feb 13;17:1749634. doi: 10.3389/fpsyg.2026.1749634 (PMC12946034; doi:10.3389/fpsyg.2026.1749634)
Supplement: Supplementary file 1 [file Table_1.docx]

Supplementary Material

# Table S1 Independent samples t-test for sex (n=683; 303 male, 380 female).

|  | | Mean equivalence t-test | |
| --- | --- | --- | --- |
| Grouping variable | Implicit variable | *t* | *p* |
| Gender | Physical Exercise | 3.816 | 0.000** |
|  | Innovative Behavior | -0.925 | 0.355 |
|  | Learning engagement | -0.563 | 0.573 |
